# Innovative integration of biometric data and blockchain to enhance ownership and trust with NFTs

**DOI:** 10.1038/s41598-025-02516-8

**Published:** 2025-05-24

**Authors:** Sung-eun Heo, Manho Kim, Wijin Kim, Jongseok Choi, Sungwon Jung, Yoogyeong Oh, Bumgyu Choi, Eunji Choi, Deokjae Heo, Sangmin Lee, Ju Hyun Park, Jinkee Hong

**Affiliations:** 1 Department of Chemical and Biomolecular Engineering, College of Engineering, Yonsei University, Seoul, 03722 Republic of Korea; 2 Department of Biomedical Science, Kangwon National University, Chuncheon, 24341 Gangwon-do Republic of Korea; 3 School of Mechanical Engineering, Chung-Ang University, 84 Heukseok-ro, Dongjak-gu, Seoul, 06974 Republic of Korea; 4 Biyard Corporation, Seocho-gu, Seoul, 06766 Republic of Korea

**Keywords:** Biological techniques, Computational biology and bioinformatics, Health care

## Abstract

Biometric data has the potential to revolutionize health analytics and pharmacology by providing personalized insights into drug efficacy and health trajectories. However, its governance presents significant ethical challenges, particularly around individual ownership and privacy. This study addresses these challenges by proposing a sustainable and ethical framework that integrates biometric data with non-fungible tokens (NFTs). We developed a customized NFT framework with advanced smart contract functionalities that enhance privacy protection and decentralized authentication of biometric data ownership. This approach ensures the secure and ethical management of digital health data while reinforcing individuals’ control over their biometric information. By leveraging cryptographic techniques for privacy protection, this framework enhances both the security and efficiency of personal health data management, offering a new perspective on ownership in digital health. Furthermore, a sustainable economic model is proposed to facilitate ethical transactions of tokenized biometric data within the NFT marketplace. The implications of this study extend beyond technology and commerce, offering valuable insights into human behavior in emerging digital economies and contributing to the creation of a more sustainable and equitable digital health ecosystem.

**Supplementary Information:**

The online version contains supplementary material available at 10.1038/s41598-025-02516-8.

## Introduction

The rapid evolution of technological innovation, particularly in artificial intelligence (AI) and machine learning (ML), is reshaping numerous sectors at an unprecedented pace. These advancements critically depend on the availability and quality of data, following the principle that higher-quality data lead to more significant insights and outcomes. Among various data types, biometric data stand out for their depth and potential utility, ranging from daily health indicators to complex genomic profiles^1^. This vast array of data enables a sophisticated understanding of health and lifestyle patterns, enhancing predictive models for pharmacological interventions and physiological simulations conducted in silico^2^.

However, the accumulation and management of such sensitive biometric data pose significant ethical and governance challenges, particularly regarding ownership rights, which are rarely conferred upon the data creators. This misalignment between data generation and proprietary rights raises serious concerns about privacy, control, and the equitable use of personal information^3^. Traditional methods—such as encryption^4^, secure hardware^5^, anonymization^6^, and zero-knowledge proofs (ZKPs)^7^—ensure privacy and security, but they primarily focus on access control rather than decentralized ownership or monetization. While ZKPs support privacy-preserving authentication, they are computationally intensive and lack data portability. Privacy-focused databases ensure regulatory compliance but depend on centralized trust, limiting user autonomy. In contrast, NFTs offer a decentralized framework enabling verifiable ownership, secure transactions, and traceability. When combined with advanced cryptographic methods, NFTs provide a scalable and privacy-enhancing solution for sensitive data management.

Non-fungible tokens (NFTs) have emerged as a transformative solution to these challenges. NFTs are unique digital tokens powered by blockchain technology, representing scarce digital assets with distinct value within a blockchain network. While previous studies have explored the integration of biometric data with NFTs^2,8–10^, they have primarily remained conceptual, focusing on theoretical discussions rather than practical implementation. In contrast, the objective of this study is to develop a secure and decentralized framework—Cell-NFT—for the ownership, management, and application of biometric data using NFT technology.

Unlike previous research focused on domains like the Internet of Vehicles (IoV)^11,12^, the Cell-NFT framework introduces innovations specifically tailored for healthcare. These innovations include standardized data management, robust ownership mechanisms, and cryptographic privacy-enhancing techniques, ensuring compliance with data protection regulations in healthcare and biotechnology.

Traditional database systems, while efficient at storing and retrieving data, rely on centralized control for access management and data integrity. This centralized approach introduces risks such as unauthorized modifications, lack of transparency, and dependence on intermediaries for trust. In contrast, blockchain technology offers a decentralized solution, where data integrity is cryptographically verifiable, and records are tamper-proof. By integrating these features, the Cell-NFT framework ensures that biometric data ownership and transactions are transparent, auditable, and resistant to manipulation.

The metadata schema for Cell-NFTs is not a simple replication of traditional database functions but is designed to leverage blockchain’s unique advantages in data security and provenance tracking. Unlike conventional databases, where data integrity depends on a central authority, blockchain-based metadata guarantees tamper-proof records, cryptographically verifiable ownership, and decentralized access control. This approach is particularly valuable in biomedical applications, where regulatory compliance, transparency, and auditability are essential. Traditional databases are vulnerable to unauthorized modifications and require extensive trust in intermediaries, whereas blockchain mitigates these risks by providing a transparent and immutable ledger of all recorded interactions. By incorporating these features, our approach offers significant improvements in data integrity, security, and long-term reliability compared to conventional storage solutions.

Our research presents a pioneering methodology that integrates biometric data into NFTs to establish robust ownership records. By tokenizing biometric information, this framework preserves individual uniqueness and represents biological identity within the digital sphere, enabling secure certification of data ownership. Biometric NFTs contain comprehensive digital life data, enabling their governance and exchange across diverse digital platforms and marketplaces. The accompanying metadata schema is designed to boost both accessibility and practical use by streamlining data sharing and transactions, thereby enhancing management workflows and reinforcing the foundation of a secure and efficient digital health infrastructure.

Tokenized biometric data have diverse applications, including use by hospitals and pharmaceutical companies. To address limitations in current NFT-based healthcare and biotechnology solutions, our framework employs a metadata schema and ecosystem designed to achieve enhanced data security, streamlined processes, and improved accessibility. This tokenization introduces societal benefits, such as improved data management security and the emergence of new industries centered on innovative digital assets. Additionally, the work underscores the concept of individuality in the digital age, presenting both challenges and opportunities at the intersection of human behavior, ethics, and societal norms.

## Results

### Issuance of Cell-NFTs using biometric data

**Fig. 1:**
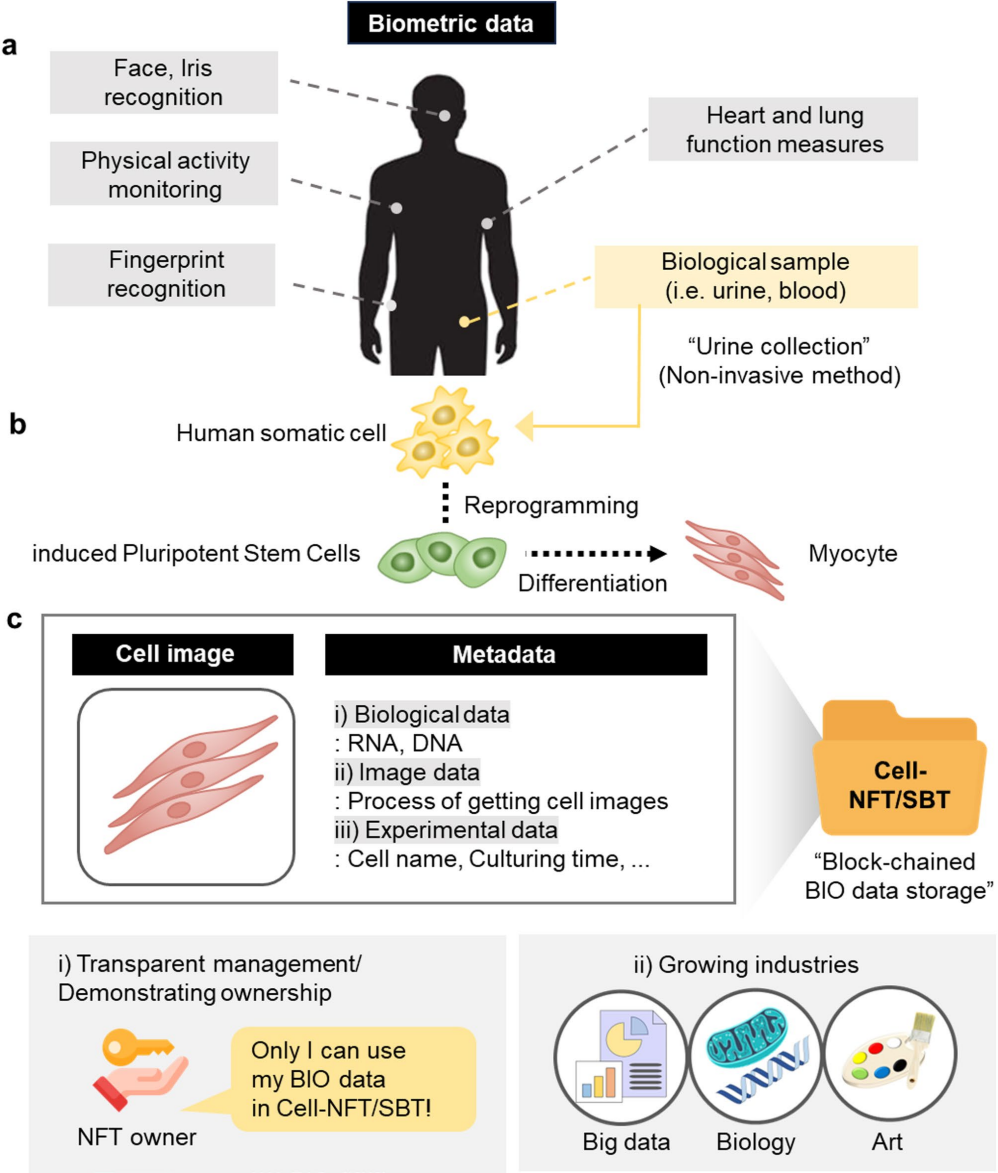
Issuance of NFTs using biometric data. (**a**) Types of biometric data derived from the human body. (**b**) Process of collecting cardiomyocytes from urine as biometric data. (**c**) Impact of NFTs created from the collected cardiomyocytes and their associated metadata.

Biometric data can be generated from various human physiological functions, including facial recognition, cardiac and pulmonary metrics, and biological sample analysis (Fig. 1a). These data are crucial for understanding individual health, identity, and physiological function. A central aim of this research is to tokenize such biological information, with a particular focus on cardiomyocytes, given their central role in heart function. By tokenizing these cells, we enable virtual ownership of an individual’s heart through NFTs. These NFTs encapsulate encrypted metadata on the blockchain, which includes key information such as genetic data, experimental details, and ownership records.

In addition to cardiomyocytes, other types of biometric data—such as those derived from various cell types (Fig. 1b)—can be tokenized using NFTs. The metadata from these cells can be utilized for identity authentication. The study protocol received ethical approval from the Institutional Review Board (IRB) of the Kangwon National University Bioethics Committee (Republic of Korea) (KWNUIRB-2024-02-008-001).

For this study, cells were derived from noninvasive, easily collectible urine samples and subsequently dedifferentiated into induced pluripotent stem cells (iPSCs) (Fig. 1b). Notably, iPSCs can differentiate into various cell types, including osteocytes, neurons, and muscle cells. Cardiomyocytes, a specific type of muscle cell, were selected for the issuance of Cell-NFTs. Given that NFTs can be produced in video format, dynamic representations of the cardiomyocytes—such as heartbeats—are provided as supplementary videos (Supplementary Videos 1 and 2), enhancing the personal connection to the tokenized data and their market value.

The encrypted metadata within these NFTs can be classified into three categories: Biological data, Image data, and Experimental data (Fig. 1c). Biological data can include unique identifiers such as cell-specific DNA and RNA sequences obtained from whole-genome sequencing (Supplementary Fig. 1). Image data consist of captured cell images that are minted as NFTs. Experimental data provide a comprehensive overview of the processes involved, such as the dates on which cells were acquired, the differentiation methods used, and the timeline of NFT creation. Within the Image and Experimental data, it is feasible to demonstrate the process of obtaining cells from an individual to establish ownership rights over the NFTs.

Cell imaging serves a dual purpose: it is both scientifically valuable and aesthetically engaging. This dual function has been recognized by companies like GE Healthcare, which organizes cell imaging competitions, as well as by renowned academic institutions such as the University of Queensland, Massachusetts Institute of Technology (MIT), and Stanford. These institutions host contests to showcase beautiful cell images, further highlighting the appeal of cellular imagery. Such collections not only attract attention for their visual impact but also provide a rich dataset, capturing the interest of the scientific community eager to extract detailed biological and experimental information.

## Reprogramming UDC into cardiomyocytes

**Fig. 2:**
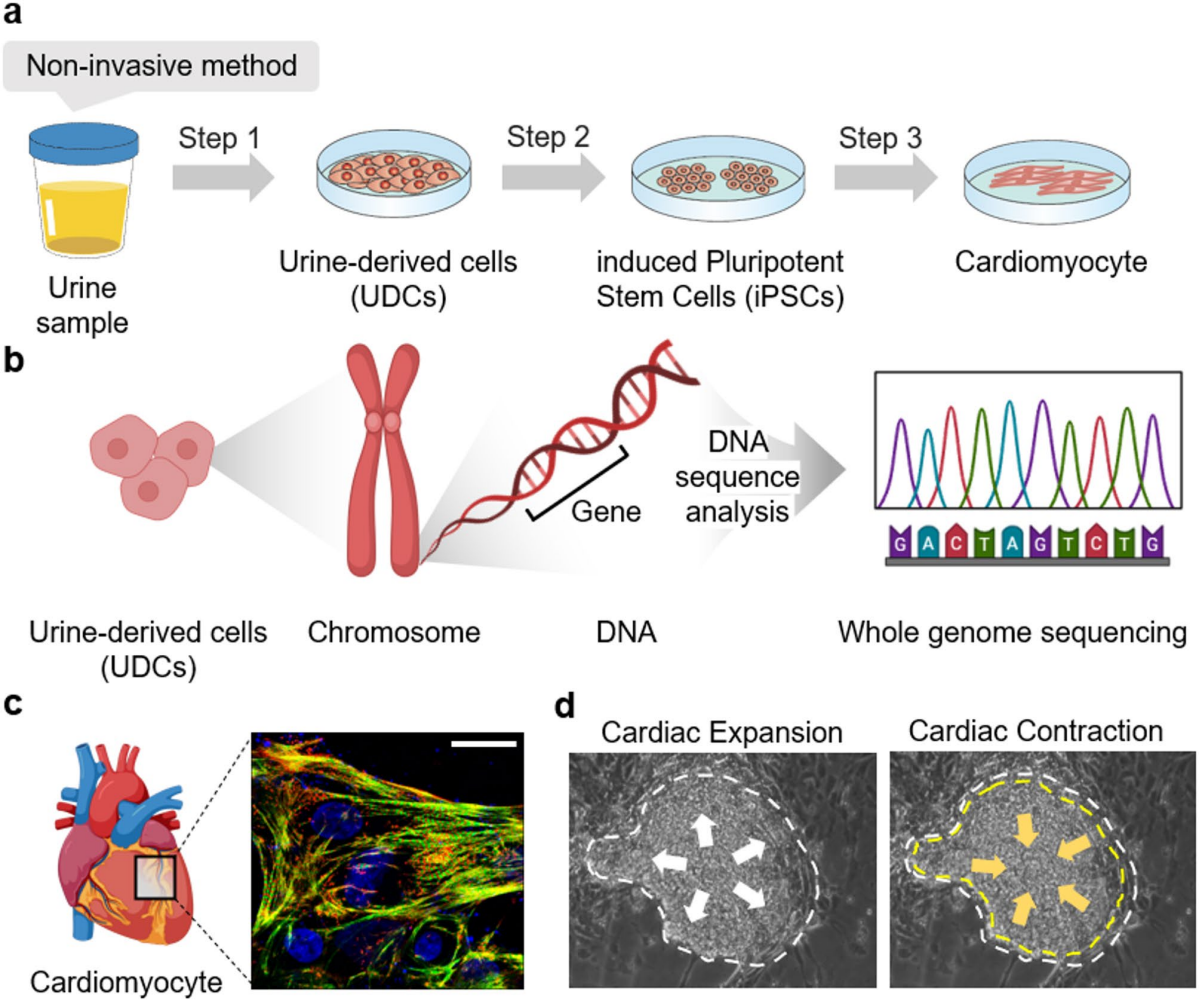
**Differentiation method and morphology of cardiomyocytes.** (**a**) Three steps for obtaining cardiomyocytes from urine. (**b**) Conceptual illustration of a WGS-based analysis of UDC-derived genetic information. (**c**) Confocal image of cardiomyocytes stained with cardiac troponin T, alpha-actinin-2, and Hoechst 33,342 (scale bar: 20 μm). (**d**) Spontaneous contraction and expansion of cardiomyocytes, mimicking a natural heartbeat.

Cardiomyocytes derived from urine samples are isolated through three critical steps, as illustrated in Fig. 2a and Supplementary Fig. 2, and detailed in previous studies^13,14^. The first step involves isolating urine-derived cells (UDCs) from urine samples. These UDCs are then reprogrammed into induced pluripotent stem cells (iPSCs) through electroporation, a non-viral technique that avoids the risks of oncogene activation associated with viral reprogramming methods^15^ (Supplementary Fig. 3). The final step involves differentiating the iPSCs into cardiomyocytes, as demonstrated in Supplementary Fig. 4.

Figure 2b illustrates a potential approach for acquiring comprehensive genetic information from UDCs using whole genome sequencing. This sequencing provides a detailed view of an individual’s unique genetic code, aiding in identity authentication^16^. Furthermore, since lifestyle and environmental factors can impact genomic data over generations^17^, storing such diverse biometric data on the blockchain (as shown in Fig. 1a) allows the analysis of their interrelationships and discovery of new insights^18,19^.

To assess the morphology of the cardiomyocytes, we captured high-resolution fluorescence images (Fig. 2c and Supplementary Figs. 5 and 6). Cardiomyocytes exhibit rhythmic contraction and expansion, mimicking the natural heartbeat at a rate of 60 beats/min (Fig. 2d and Supplementary Videos 1 and 2).

The implications of these findings within the NFT space are profound. Integrating human life data into the metaverse marks a pioneering development, garnering significant interest from potential NFT participants. This integration serves two important purposes: it helps alleviate apprehensions individuals may have about the metaverse while also offering a unique, personal way to preserve and memorialize the biological data of loved ones in a virtual space. This allows for the celebration and preservation of life, even in the digital realm.

## NFT issuance and metadata from the extracted cells

**Fig. 3:**
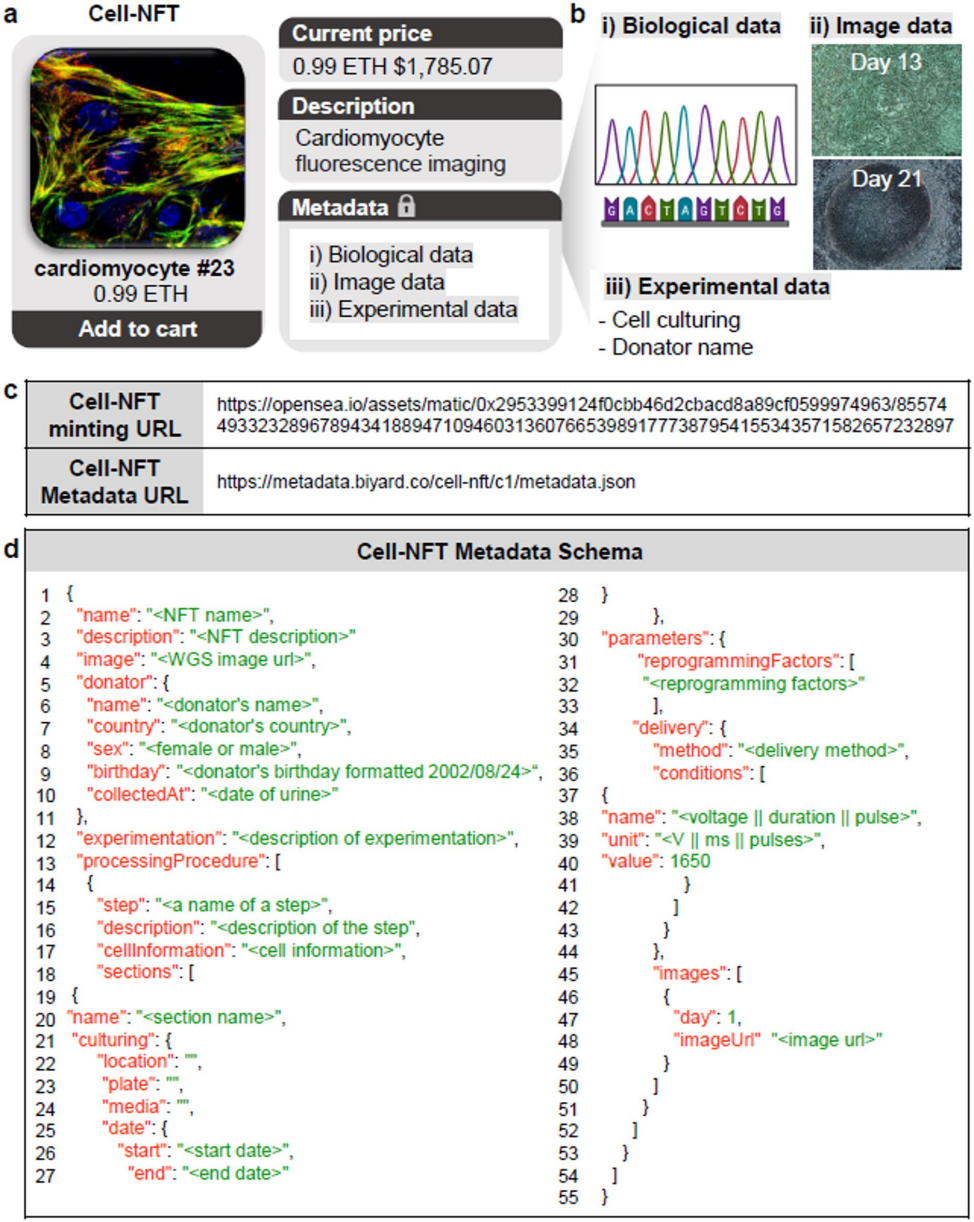
**Metadata in an issued Cell-NFT.** (**a**) Illustration of an issued Cell-NFT with three categories of metadata. (**b**) Detailed breakdown of each metadata category. (**c**) URL of the minted Cell-NFT containing cardiomyocytes and its associated metadata. (**d**) Metadata schema for the Cell-NFT with cardiomyocytes.

The fluorescence image of the cardiomyocyte shown in Fig. 2c was minted as a unique digital token, termed as Cell-NFT. This token encapsulates extensive information, with its value being inherently linked to the intrinsic attributes and cell origin. For example, a cell derived from a notable individual could command a higher value. As illustrated in Fig. 3a, these Cell-NFTs are available for acquisition in the contemporary metaverse marketplace using digital currencies such as Ethereum^20^.

Ethereum utilizes the environmentally sustainable Proof of Stake (PoS) consensus mechanism^21–23^, which significantly reduces the energy consumption typically associated with mining. This eco-friendly approach offers substantial environmental benefits, particularly for large-scale applications. In contrast, Proof of Work (PoW) mining requires considerable electrical energy and computational resources for block creation and verification, resulting in enhanced security. Moreover, PoW’s decentralized nature fosters a secure, independent network. As such, many blockchain platforms suitable for the creation of these added-value tokens will emerge, with the most effective methods likely evolving through collaboration with major projects like Bitcoin, Bitmobick, and Ethereum.

Similar to conventional NFTs, each Cell-NFT is accompanied by a descriptive section that provides potential investors with a summary of the tokenized cell, thereby enhancing its appeal. Crucially, the metadata, which serve as the digital fingerprint of the token, are encrypted and securely embedded within the NFT via blockchain technology.  As illustrated in Fig. 3b, metadata are categorized into three domains: “Biological data,” “Image data,” and “Experimental data.” The integration of these metadata categories is fundamental to the minting of each Cell-NFT.

Once minted, Cell-NFTs are made available on OpenSea, a prominent NFT marketplace. These tokens hold value beyond marketplace transactions; they also have practical applications in sectors like healthcare and pharmaceuticals, depending on their intended purpose. URLs linking to these NFTs are displayed in Fig. 3c. Although OpenSea offers a public-facing platform, it ensures the privacy of metadata by concealing its detailed content. Only the legitimate owner of the Cell-NFT can access the complete metadata, which is structured as shown in Fig. 3d.

## Metadata Schema of Cell-NFT

The metadata of a Cell-NFT comprise various types of cell-related information, organized within a structured schema as shown in Fig. 3d. This schema includes sections such as “name,” “description,” “image,” “donator,” “experimentation,” and “processing procedure,” each detailing specific aspects of the cell. Supplementary Fig. 7 provides detailed explanations of each section. Additionally, comprehensive metadata associated with the Cell-NFT can be accessed through the designated metadata URLs in Fig. 3c and Supplementary Figs. 8 and 9.

**Fig. 4:**
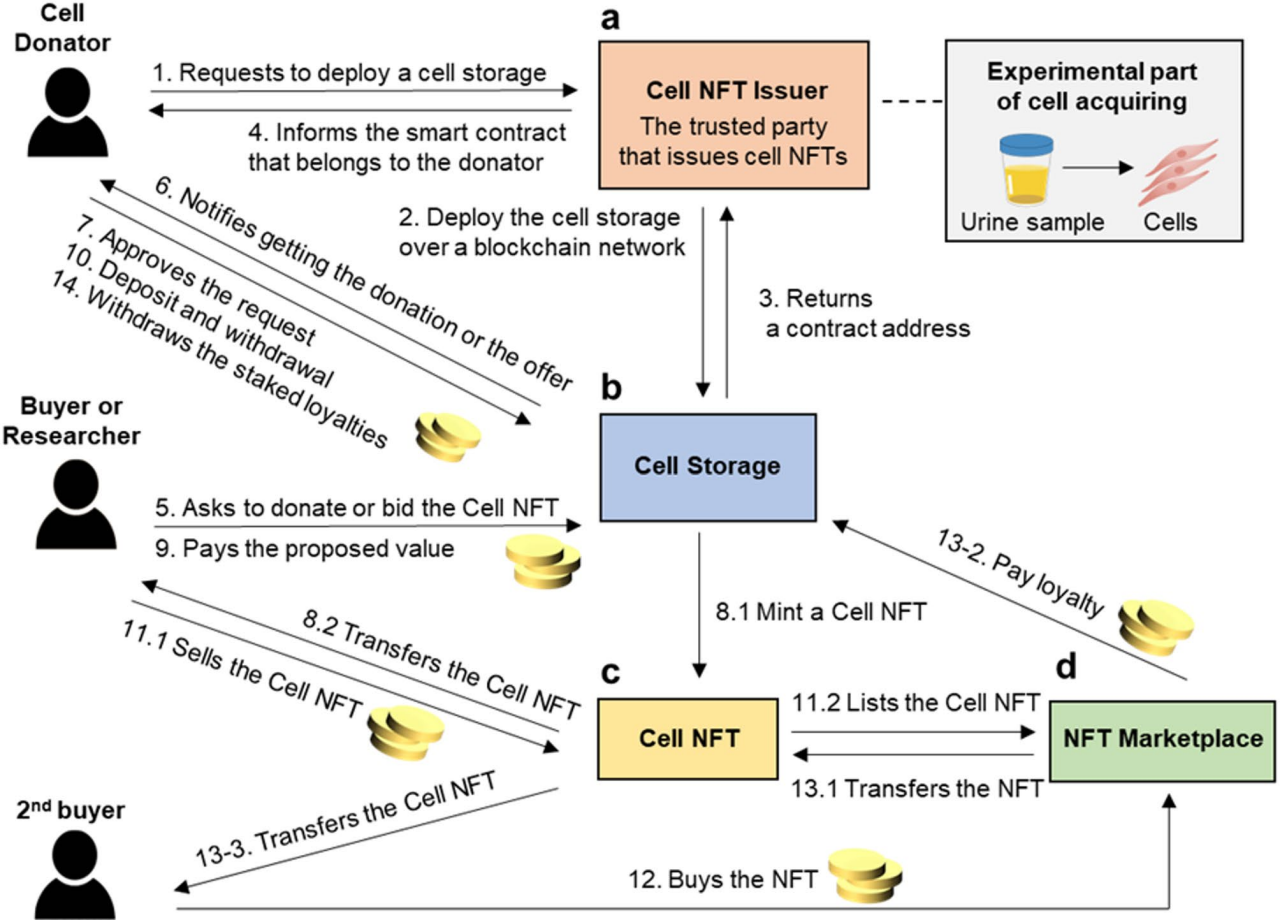
**Cell-NFT ecosystem:** (**a**) Cell NFT Issuer. (**b**) Cell Storage. (**c**) Cell-NFT. (**d**) NFT Marketplace.

## Cell-NFT ecosystem

The Cell-NFT ecosystem introduces a groundbreaking approach to the representation and transaction of cellular data, as illustrated in Fig. 4. This ecosystem enables the value and ownership of biological data to be represented as NFTs. In this system, ownership of a Cell-NFT not only confers biological data but also confers tangible economic value, making the NFT tradable in various NFT marketplaces.

As shown in Fig. 4a, when a cell donor wishes to tokenize their biological data, they request a Cell-NFT issuer to facilitate the creation of Cell Storage. The issuer, which can be an individual or institution, plays a critical role in validating the NFT prototype through consensus from a pool of trusted issuers. Upon issuance, Cell Storage provides a unique contract address to the Cell-NFT issuer and updates the donor’s affiliated smart contract.

If a buyer or researcher expresses interest in the cell’s data, Cell Storage acts as an intermediary, alerting the donor to any incoming offers or bids (Fig. 4b). Upon the donor’s approval, Cell Storage proceeds to mint the Cell-NFT (Fig. 4c). The minted NFT is then transferred to the interested buyer or researcher following payment. A real-world scenario from a researcher’s perspective illustrates how Cell-NFTs can be minted, traded in marketplaces, and utilized in scientific experiments.

This ecosystem ensures the donor retains control over the funds held in the Cell Storage, allowing them to manage withdrawals and deposits at their discretion. A key feature of the ecosystem is its flexibility for secondary market trading. After an initial purchase, the buyer or researcher can list the Cell-NFT on an open marketplace (Fig. 4d), allowing secondary buyers to participate in the transaction. However, to protect the donor’s rights, the ecosystem grants the donor the option to restrict secondary sales to authorized entities or collect loyalty fees from subsequent trades of their Cell-NFT.

In essence, the Cell-NFT ecosystem merges biotechnology with digital assets, offering a new paradigm for bio-data issuance, ownership, and exchange.

## Discussion

Our exploration of the convergence between biometric data and blockchain technology, culminating in the creation of Cell-NFTs, represents a transformative step in the digitalization of biological assets. The Cell-NFT model offers an innovative solution by using blockchain to manage access permissions for biometric data, ensuring individual autonomy, transparent governance, and regulatory compliance.

While previous research has primarily conceptualized the application of biometric data to NFTs^2,8–10^, our study provides actionable proposals for its implementation. We have developed an integrated NFT system that overcomes prior challenges related to data security, increased risks, cumbersome processes, and limited user engagement.

Recent studies emphasize blockchain’s potential in improving financial ecosystems and fostering societal trust through decentralized systems^24^. Building on these insights, the Cell-NFT framework not only advances biometric data management but also contributes to broader economic efficiency and blockchain innovation. This reinforces the social value of secure, self-sovereign digital identity solutions, particularly in healthcare.

A key element in this transformation is the valuation of biometric data, which varies based on factors like rarity, sensitivity, and application. Establishing a robust valuation framework that accounts for data scarcity, its significance in healthcare, and its research potential is essential. Ensuring fair compensation for individuals while promoting ethical trading practices is crucial for fostering a sustainable and transparent Cell-NFT marketplace.

The implications for medical diagnostics and therapeutics demand careful consideration of ethical and regulatory frameworks. As NFT adoption grows, legal standards continue to evolve, requiring ongoing compliance. Our framework does not facilitate the commercialization or unrestricted trading of biometric data but functions as a cryptographic mechanism for access control, ensuring individual ownership and autonomy. It enforces explicit, revocable consent while adhering to ethical guidelines that prioritize privacy and prevent unauthorized use. By design, the system prevents biometric data from being commodified, reinforcing responsible and compliant management.

Recent studies highlight the cybersecurity concerns and legal complexities of blockchain regulations across jurisdictions^25^. These studies underscore the need for alignment with NIST and ISO standards to ensure compliance. The Cell-NFT framework strengthens trust, security, and legal compliance in biometric data management by aligning with global regulations. It enables individuals to maintain control over their data via NFTs while adhering to laws such as HIPAA^26,27^, GDPR^28^, and the Australian Privacy Act^29^. These regulations require explicit consent, breach reporting, and user rights to access, modify, or delete data. Ethical standards like the Australia’s National Statement^30^ and global standards like the Declaration of Helsinki^31^ further emphasize informed consent, confidentiality, and responsible data handling.

Ensuring ethical and legal compliance in NFT-based biometric data management is critical, especially in the context of data breaches, misuse, and privacy violations. Key risks include unauthorized access, data manipulation, and accidental loss, which can compromise privacy. The Cell-NFT framework mitigates these risks through encryption, multi-factor authentication (MFA), and real-time monitoring. Smart contracts enforce compliance, while automated audit trails and breach notification mechanisms ensure transparency and regulatory adherence (GDPR, HIPAA). These strategies collectively strengthen trust, security, and accountability in decentralized biometric data management.

To safeguard ethical data usage further, we propose using smart contracts to enforce data usage conditions. These contracts would ensure secure, automated transactions and transfer ownership of Cell-NFTs only when ethical standards are met. By implementing these measures, biometric NFTs align with global privacy regulations, ethical standards, and emerging regulatory requirements for digital assets. Compliance with regulations such as GDPR, HIPAA, and local data protection laws is essential to responsibly manage biometric data. Trust in the Cell-NFT framework is reinforced through blockchain’s immutable data storage, verifiable ownership via NFTs, and decentralized authentication via smart contracts. This trust is further bolstered by cryptographic security, decentralization, and blockchain transparency, which protect the integrity and confidentiality of biometric data.

Market dynamics, including demand, supply chains, and secondary sales, will significantly impact the value and liquidity of Cell-NFTs. Pricing models will incorporate factors like data uniqueness, demand for health data, and research potential. Ensuring fair trade and robust market mechanisms will be essential for the widespread adoption of the system.

A formal threat model has been developed to address risks such as data leakage, unauthorized access, smart contract vulnerabilities, and Sybil attacks. To mitigate these risks, the Cell-NFT framework employs encryption, zero-knowledge proofs (ZKP), and role-based access control (RBAC) to ensure data security and integrity.

Ensuring the long-term sustainability of the Cell-NFT system requires continuous optimization, adaptive strategies, and efficient user management. A dynamic metadata structure supports regular updates, while a user-friendly interface with role-based access controls enhances both security and accessibility. To address scalability, our framework incorporates Layer 2 solutions like rollups and sidechains, improving transaction throughput while reducing gas fees and computational overhead. A hybrid storage model securely stores biometric data off-chain, recording only cryptographic proofs on-chain to minimize storage costs and enhance blockchain efficiency. Additionally, off-chain processing and decentralized storage (e.g., IPFS, Arweave) ensure data integrity, regulatory compliance, and tamper-proof record-keeping. These improvements collectively enhance performance, scalability, and cost-effective decentralized access control in an evolving technological landscape.

While we acknowledge the limitation of not conducting direct experimental validation, this literature-driven comparative analysis provides a solid foundation for assessing the feasibility and potential impact of Cell-NFT. Traditional databases typically process transactions at approximately 500 TPS, while Cell-NFT, using Layer 2 scaling solutions, achieves 50 TPS while enhancing security. Centralized databases offer lower storage costs, whereas Cell-NFT mitigates high on-chain storage expenses by leveraging IPFS for off-chain data management. Furthermore, blockchain ensures immutable, decentralized security, whereas traditional databases rely on centralized administration, making them more susceptible to unauthorized modifications and security breaches.

Future work will focus on metadata standardization and retrieval within the Cell-NFT framework. The JSON-based schema will ensure interoperability, adhering to RDF and DCAT standards. Cross-platform adoption will be facilitated through integration with RESTful APIs, OData protocols, and schema validation. Key attributes such as “name,” “description,” and “experimentation” will support interoperability testing and data transformation, enhancing data accessibility, security, and regulatory compliance. Additionally, we plan to collaborate with healthcare institutions and industry partners to conduct pilot studies, evaluating the feasibility, security, and regulatory compliance of the Cell-NFT framework. These empirical investigations will bridge the gap between theoretical design and real-world implementation, ensuring scalability and adoption in secure data transactions.

## Methods

### Collection of human urine from the participants

Urine samples were collected from individual donors in compliance with the ethical guidelines outlined in the 64 th World Medical Association Declaration of Helsinki. The procedure was approved by the Institutional Review Board (IRB) at the Kangwon National University Bioethics Committee (Republic of Korea) (KWNUIRB-2024-02-008-001). Informed consent was obtained from all donors before sample collection. A total of four human donors participated in this study (three male, one female), though the gender of the donors was not relevant to the outcomes of the study. Human urine samples were pooled to isolate epithelial cells, with the results not being influenced by the characteristics of the individual participants. The participation was voluntary, and only self-reported healthy individuals were included as eligible donors. Each donor provided between 250 and 300 mL of urine, which was stored in sterilized laboratory tubes at −4 °C until further processing.

### Derivation of UDCs from urine samples

Epithelial cells were isolated from urine samples as described previously^13^. To isolate UDCs, the primary medium (PM) was prepared by combining DMEM/F12 (Thermo Fisher Scientific), 10% Fetal Bovine Serum (FBS, Welgene), REGMTM SingleQuotsTM supplements (Lonza), and 100 µg/mL Primocin^®^ (InvivoGen). The REMC medium was prepared by mixing RE medium and MC medium in a 1:1 ratio. The RE medium consisted of REGMTM basal medium (Lonza), 10% FBS (Welgene), REGMTM SingleQuotsTM supplements (Lonza), and 100 µg/mL Primocin^®^ (InvivoGen), while the MC medium contained DMEM high glucose (Thermo Fisher Scientific), 10% FBS (Welgene), 1X MEM GlutaMaxTM (Thermo Fisher Scientific), 1X MEM Non-Essential Amino Acids (Thermo Fisher Scientific), 100 µg/mL Primocin^®^ (InvivoGen), 5 ng/mL Basic Fibroblast Growth Factor (bFGF, Peprotech), 5 ng/mL Platelet-Derived Growth Factor (PDGF)-AB (Peprotech), and 5 ng/mL Epidermal Growth Factor (EGF, Peprotech). Washing buffer was prepared with PBS supplemented with 100 µg/mL Primocin^®^ (InvivoGen).

A 12-well plate was coated with either Matrigel or 0.1% gelatin solution for over an hour to facilitate cell adherence before urine collection. A sample of 250–300 mL was collected and centrifuged at 500 ×g for 10 min at room temperature. The sample was washed with the washing buffer, followed by a second centrifugation and washing step. After aspirating the supernatant, the resulting pellet was resuspended in 3 mL of primary medium containing 10 µM ROCK inhibitor and transferred to a single well of a 12-well plate, where the cells were incubated at 37 °C (day 0). On days 2 and 3, 1 mL of primary medium was added to the well each day. On day 4, most of the medium was aspirated, leaving 1 mL, to which 1 mL of REMC medium was added. From day 4 onward, 1 mL of the existing medium was removed and replaced daily with 1 mL of fresh REMC medium. Upon observing cell colony formation around day 9, the medium was replaced with fresh REMC medium. Upon reaching 70% confluency, the cells were detached using 0.25% Trypsin/EDTA and subcultured onto a new plate coated with Matrigel or 0.1% gelatin.

### Reprogramming UDCs to iPSCs

UDCs were reprogrammed to iPSCs as previously described^13^. The electroporation medium (EM) was prepared using DMEM (Thermo Fisher Scientific) supplemented with 10% FBS (Welgene). Essential 8™ Medium (Gibco) and Essential 8™ Supplement (50X) were used to prepare the E8 medium. To enhance the viability of iPSCs during reprogramming, a ROCK inhibitor (Cayman) was included. The Yamanaka factors pCXLE-hOCT3/4-shp53-F, pCXLE-hSK, and pCXLE-hUL (all from Addgene) were utilized to reprogram UDCs into iPSCs.

Before reprogramming, either Matrigel or 0.1% gelatin solution was applied to a 35 mm dish for over an hour. The Yamanaka factors (hOCT3/4, hSK, and hUL) were prepared at a concentration of 1 µg/µL. The UDCs were detached using 0.25% Trypsin/EDTA, followed by centrifugation at 300 ×g for 3 min. After aspirating the supernatant, the cells were counted for electroporation, with 6 × 10^5^ cells required per 100 µL tip. The cells were then resuspended in 100 µL R buffer for electroporation. The plasmids containing the reprogramming factors (hOCT3/4, hSK, and hUL) were introduced into the cells through electroporation at 1650 V, 10 ms, and 3 pulses. After electroporation, the cells were transferred to a 35 mm dish containing EM (day 0), and the cultures were maintained in E8 medium with daily medium changes. After 14 days, cell morphology transitioned from elongated to rounded, indicating the formation of iPSCs. The areas with rounded morphology were isolated by mechanical passaging, and a ROCK inhibitor was added to the E8 medium to ensure stable passaging. Stable iPSC lines were established after several rounds of mechanical passaging.

### Differentiating cardiomyocytes from iPSCs

Cardiomyocyte differentiation from iPSCs was carried out as described previously^14^. To differentiate iPSCs into cardiomyocytes, RPMI Media (Gibco) and B-27 Supplements, both with and without insulin (Gibco), were used to prepare the cardiomyocyte differentiation media. Insulin is known to inhibit cardiac differentiation during the initial five days of iPSC differentiation^14^. Consequently, RPMI media supplemented with insulin-free B-27 was used up to day 5, after which the medium was switched to RPMI with B-27 containing insulin. To promote differentiation into the mesodermal lineage, a GSK3 inhibitor, CHIR 99021 (Sigma Aldrich), and a Wnt ligand production inhibitor, IWP (Sigma Aldrich), were added to the media. For single-cell dissociation of iPSCs, Accutase (Sigma Aldrich) was employed.

Before seeding, iPSCs were coated on a 12-well plate with either Matrigel or a 0.1% gelatin solution for 1 h to enhance cell adherence. The iPSCs were then dissociated into single cells using Accutase for 8 min at 37 °C. The dissociated cells were collected and centrifuged at 200 ×g for 3 min. Approximately 0.5–1.5 × 10^6^ cells per well were seeded into the 12-well plate with E8 media containing ROCK inhibitor (day 4). The media were changed daily until day 0 when the cells reached 85–90% confluency. On day 0, the medium was aspirated and replaced with RPMI containing insulin-free B-27 and 12 µM CHIR 99,021. On day 1, the media were changed to RPMI with insulin-free B-27. On day 3, 1 mL of RPMI with insulin-free B-27 and 5 µM IWP2 was added to the medium. The media were then replaced on day 5 with RPMI containing insulin-free B-27, and on day 7, the medium was switched to RPMI with B-27 containing insulin. From this point onward, media changes were performed every 3 days using RPMI with insulin-containing B-27 (Supplementary Fig. 4).

Successful differentiation was confirmed by observing cardiomyocyte beating, a hallmark of cardiac differentiation, as shown in Supplementary videos 1 and 2. Figures 2b and c and 3b, and Supplementary Fig. 1 were created in BioRender. Heo, S. (2025) BioRender URL.

### Immunostaining analysis

For immunocytochemistry, cells were fixed with 4% paraformaldehyde (PFA) for 15 min and washed with phosphate-buffered saline (PBS). They were then permeabilized with 0.25% Triton X-100 in PBS for 30 min, followed by a PBS wash. After blocking with 3% bovine serum albumin (BSA) in PBS, the cells were washed with 0.1% Tween 20 in PBS (PBS-T). The cells were then incubated overnight at 4 °C with anti-cTNT (Santa Cruz) and anti-ACTN2 (ABclonal) primary antibodies. After incubation, the cells were washed with PBS-T, and Alexa Fluor 488- and 594-conjugated secondary antibodies (Thermo Fisher Scientific, Waltham, MA, USA) were applied at 20 °C for 1 h. The cells were then washed again with PBS-T, followed by incubation with 1 µg/mL Hoechst 33,342 in PBS for 1 h, and a final wash with PBS. Fluorescence was observed using confocal laser scanning microscopy (Carl Zeiss, Oberkochen, Germany) (Supplementary Figs. 5 and 6).

### Contraction and expansion of cardiomyocytes

Cardiomyocyte contraction and expansion images were captured using the video function of the microscope. While cardiomyocytes were visibly beating without the aid of a microscope, we used the microscope to more precisely observe the beating patterns. A single beat was defined as a full cycle of contraction and expansion. The number of contraction-expansion cycles within a 60-s observation period was counted to determine the frequency of the cardiomyocytes.

## Electronic supplementary material

Below is the link to the electronic supplementary material.

Supplementary Material 1

Supplementary Material 2

Supplementary Material 3

## Data Availability

All structured data supporting the findings of this study are provided in the published article and its supplementary files.
