# Supplementary material for: Innovative integration of biometric data and blockchain to enhance ownership and trust with NFTs
Source: Sci Rep. 2025 May 24;15:18050. doi: 10.1038/s41598-025-02516-8 (PMC12102342; doi:10.1038/s41598-025-02516-8)
Supplement: Supplementary file 1 — Supplementary Material 1 [file 41598_2025_2516_MOESM1_ESM.docx]

**Supplementary Information**

**Innovative Integration of Biometric Data and Blockchain to Enhance Ownership and Trust with NFTs**

Sung- eun Heo^a^, Manho Kim^b^, Wijin Kim^b^, Jongseok Choi^d^, Sungwon Jung^a^, Yoogyeong Oh^a^, Bumgyu Choi^a^, Eunji Choi^a^, Deokjae Heo^c^, Sangmin Lee^c,*^, Ju Hyun Park^b,*^, Jinkee Hong^a,*^

^*^Corresponding author: Jinkee Hong, Ph. D. (Email: jinkee.hong@yonsei.ac.kr)

^*^Corresponding author: Juhyun Park, Ph. D. (Email:  [juhyunpark@kangwon.ac.kr](mailto:juhyunpark@kangwon.ac.kr))

^*^Corresponding author: Sangmin Lee, Ph. D. (Email: [slee98@cau.ac.kr](mailto:slee98@cau.ac.kr))

**Table of Contents**

Supplementary Figure 1. Conceptual illustration of whole genome sequencing workflow

Supplementary Figure 2. Generation of human cardiomyocyte from urine sample and morphology

**Supplementary Figure 3.** Process of reprogramming UDC to make iPSC

**Supplementary Figure 4.** Process of iPSC to cardiomyocyte differentiation

**Supplementary Figure 5.** Immunostaining analysis of iPSC

**Supplementary Figure 6.** Immunostaining analysis of cardiomyocyte

**Supplementary Figure 7.** Specific description of Cell-NFT metadata schema

**Supplementary Figure 8.** The metadata of the actual issued Cell-NFT

**Supplementary Figure 9.** Cell-NFT Metadata example

**Video Legends**

**Supplementary Video 1.** Beating of UDC derived cardiomyocyte

This video demonstrates the spontaneous beating activity of cardiomyocytes derived from urine-derived cells (UDCs). The rhythmic contractions indicate successful differentiation and functional maturation of the cardiomyocytes.

**Supplementary Video 2.** Beating of UDC derived cardiomyocyte

This video demonstrates the spontaneous beating activity of cardiomyocytes derived from urine-derived cells (UDCs). The rhythmic contractions indicate successful differentiation and functional maturation of the cardiomyocytes.


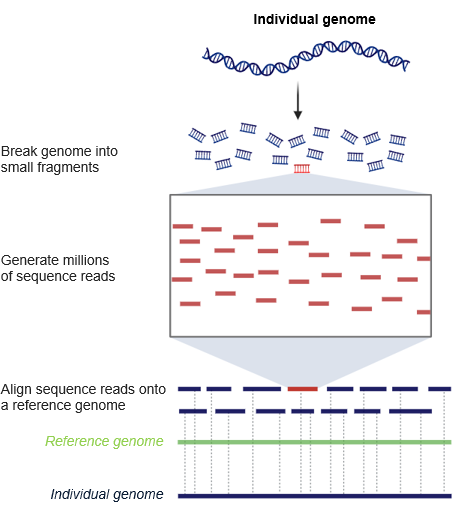


Supplementary Figure 1. Conceptual illustration of whole genome sequencing workflow.


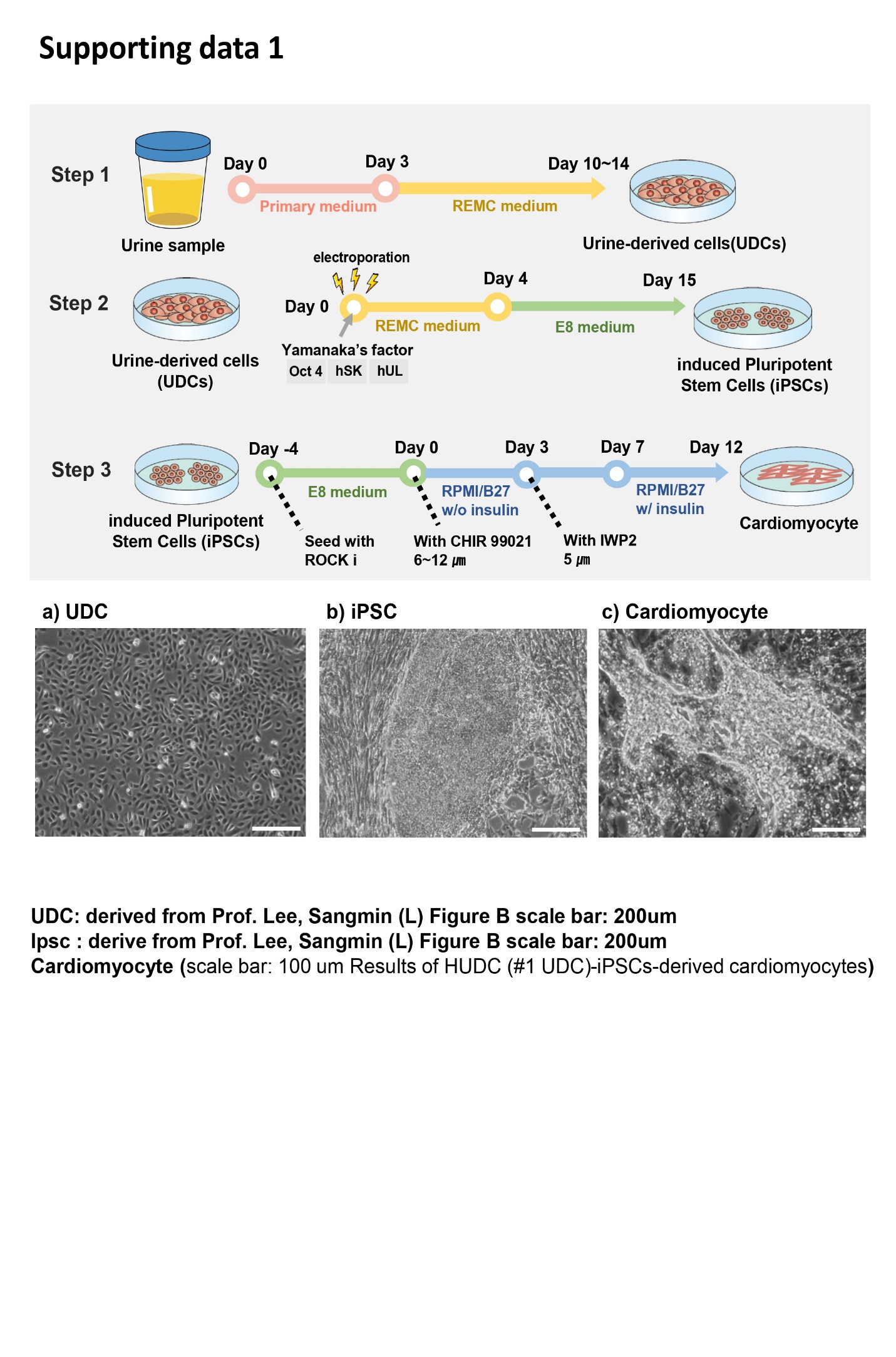


Supplementary Figure 2. Generation of human cardiomyocyte from urine sample and morphology. a) Urine-derived cells(UDCs)(Scale bar: 200um); b) induced Pluripotent Stem Cells(iPSCs) (Scale bar: 200um); c) Cardiomyocyte(Scale bar: 100um)


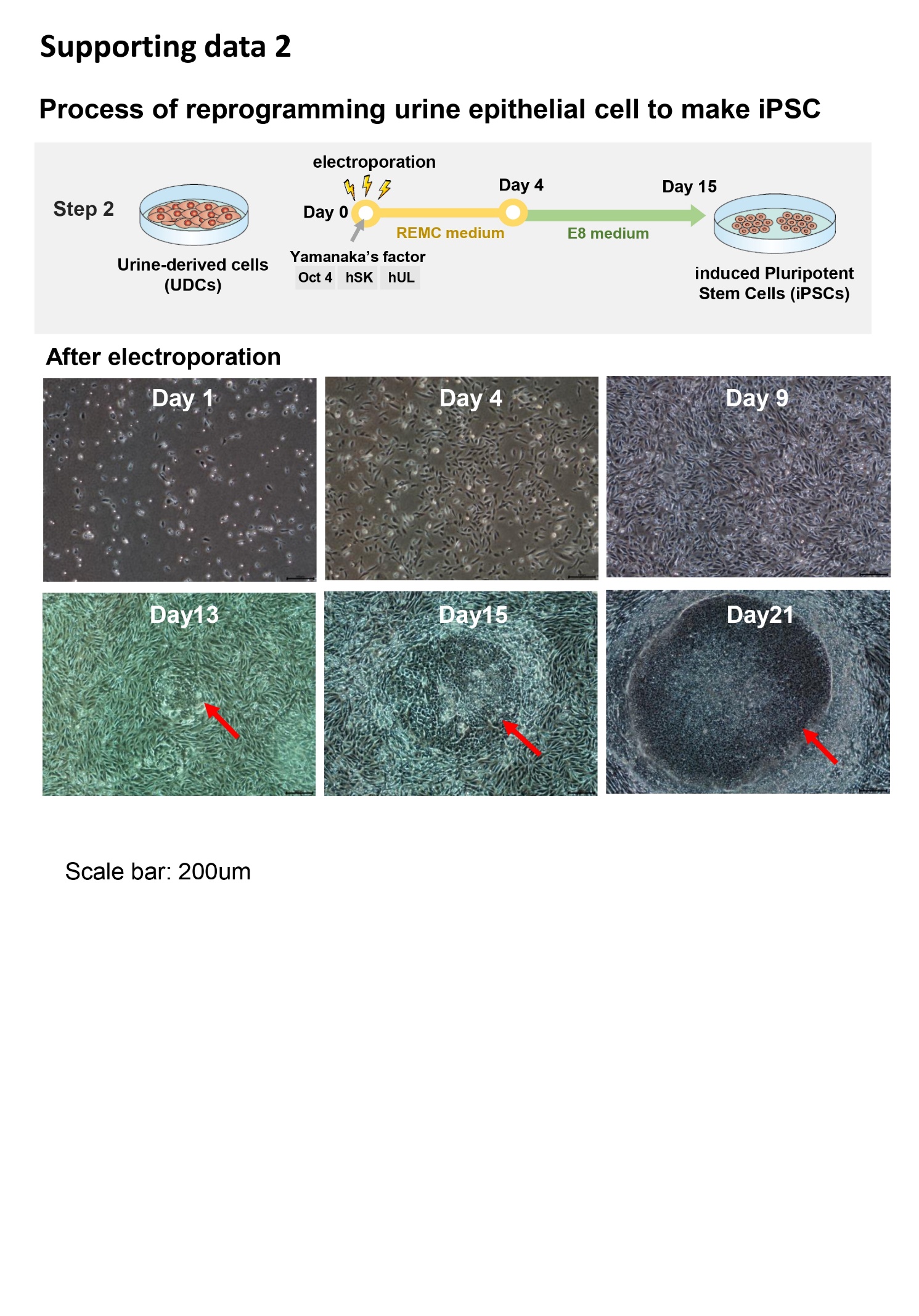


Supplementary Figure 3. Process of reprogramming UDC to make iPSC (Scale bar: 200um). Mesenchymal-epithelial transition(MET) begins at Day13, and make a border line at Day 21.


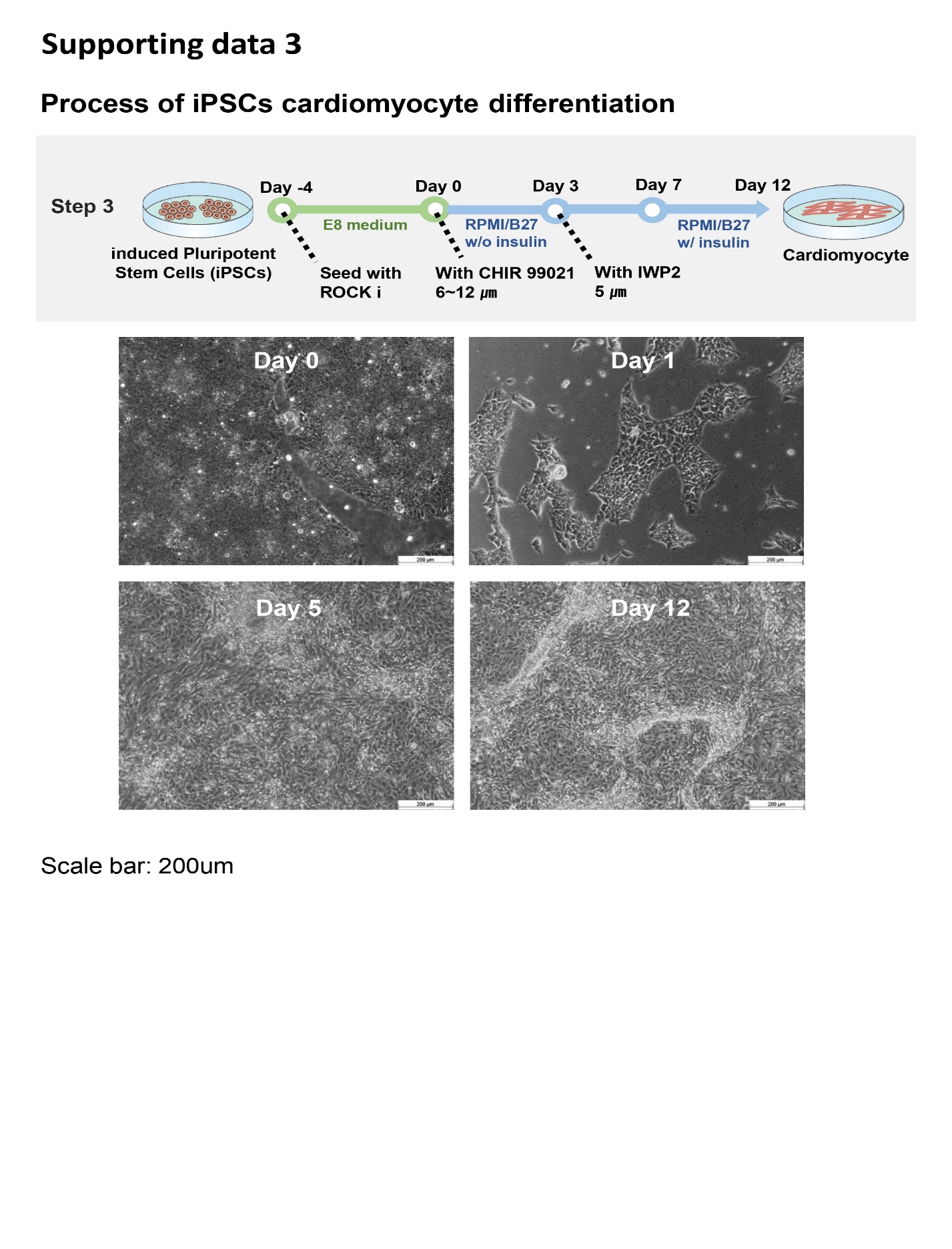


Supplementary Figure 4. Process of iPSC to cardiomyocyte differentiation (Scale bar: 200um).


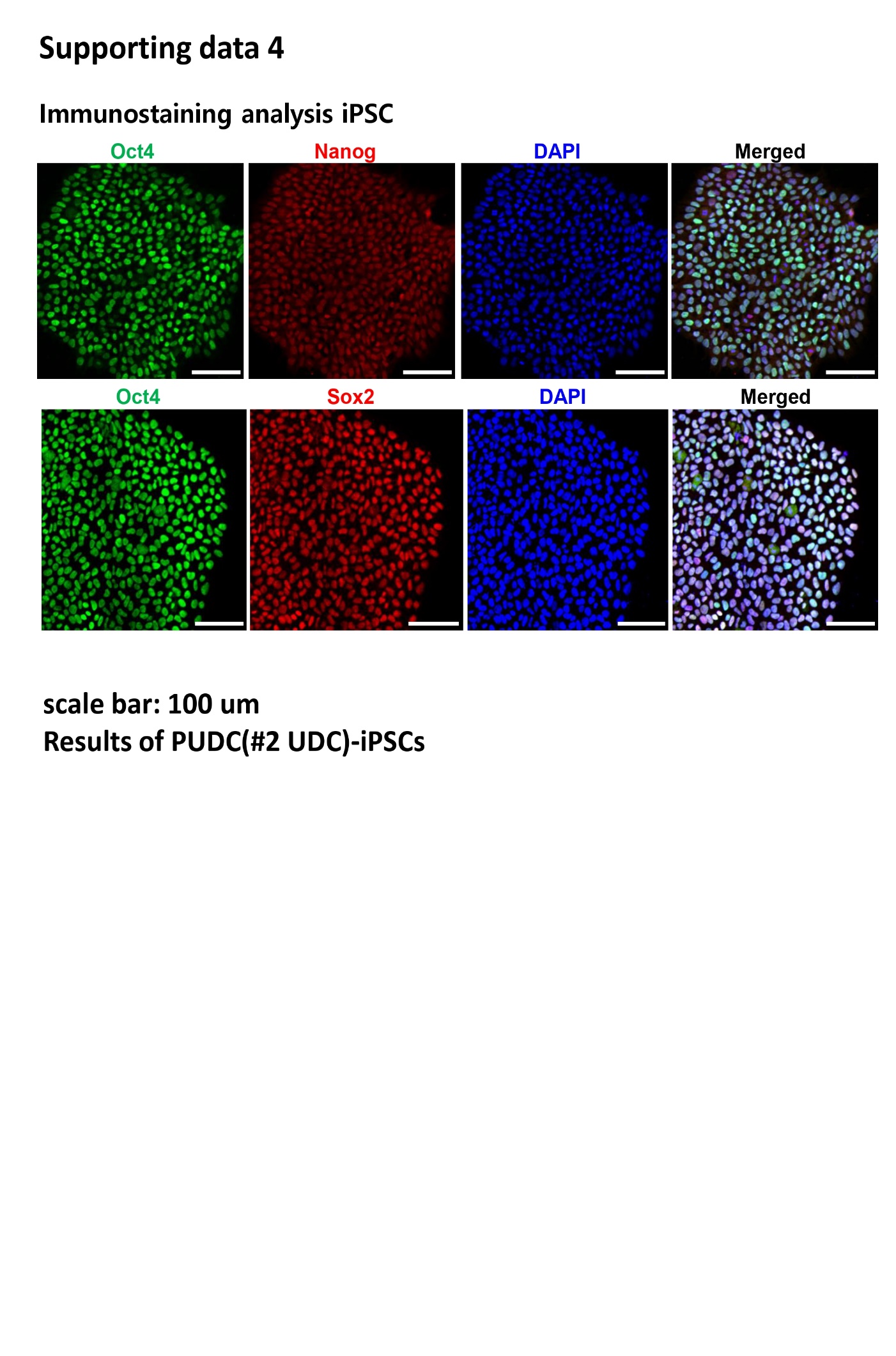


Supplementary Figure 5. Immunostaining analysis of iPSC (Scale bar: 100um).


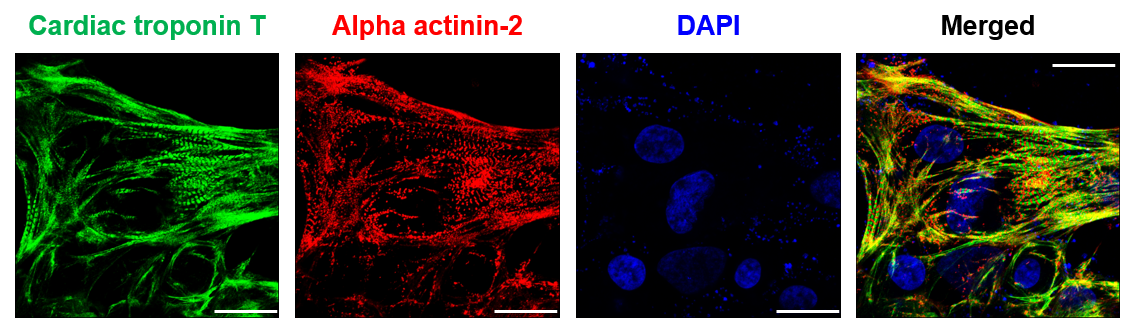


Supplementary Figure 6. Immunostaining analysis of cardiomyocyte (Scale bar: 20um).

**
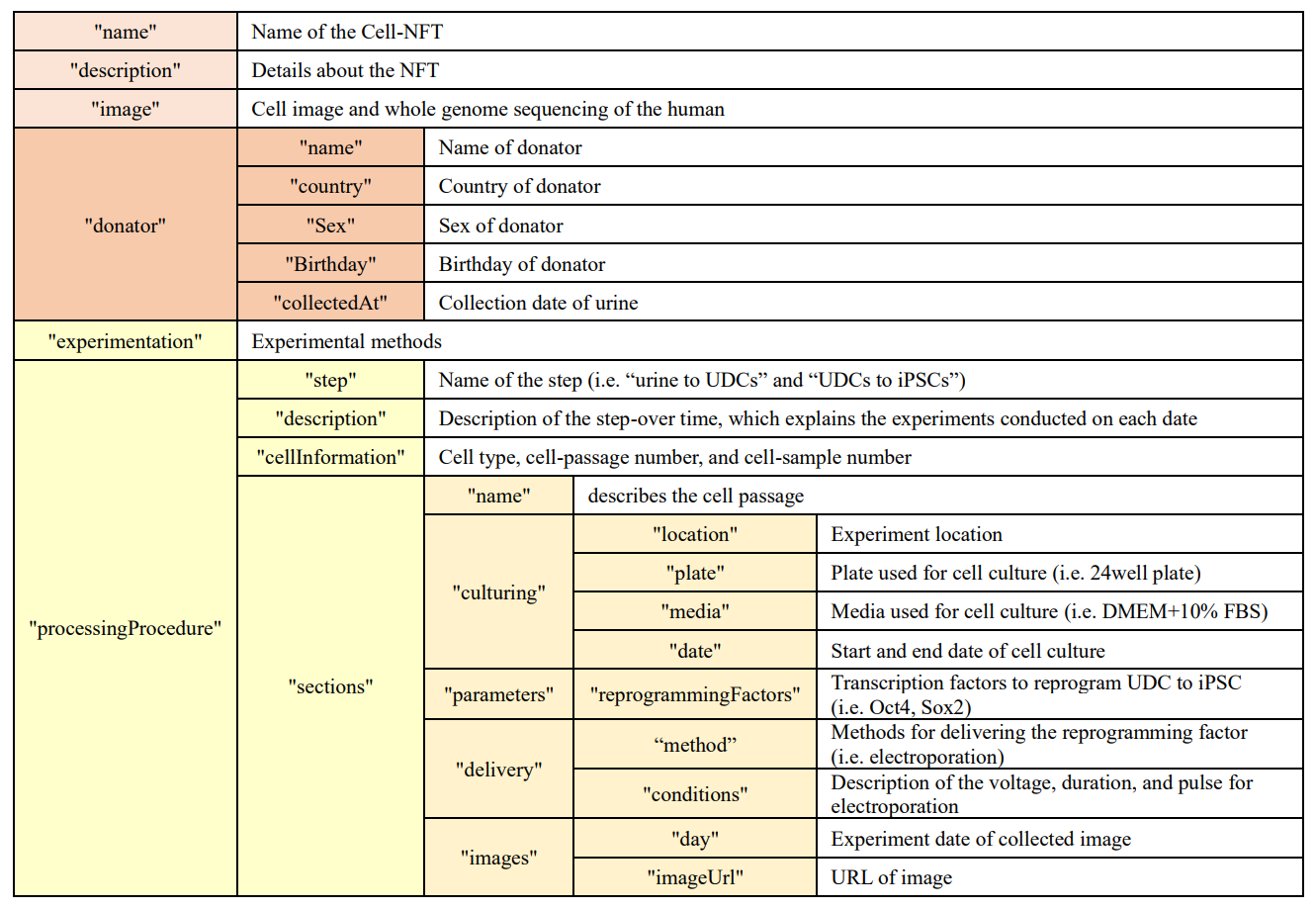
**

**Supplementary Figure 7.** Specific description of Cell-NFT metadata schema.


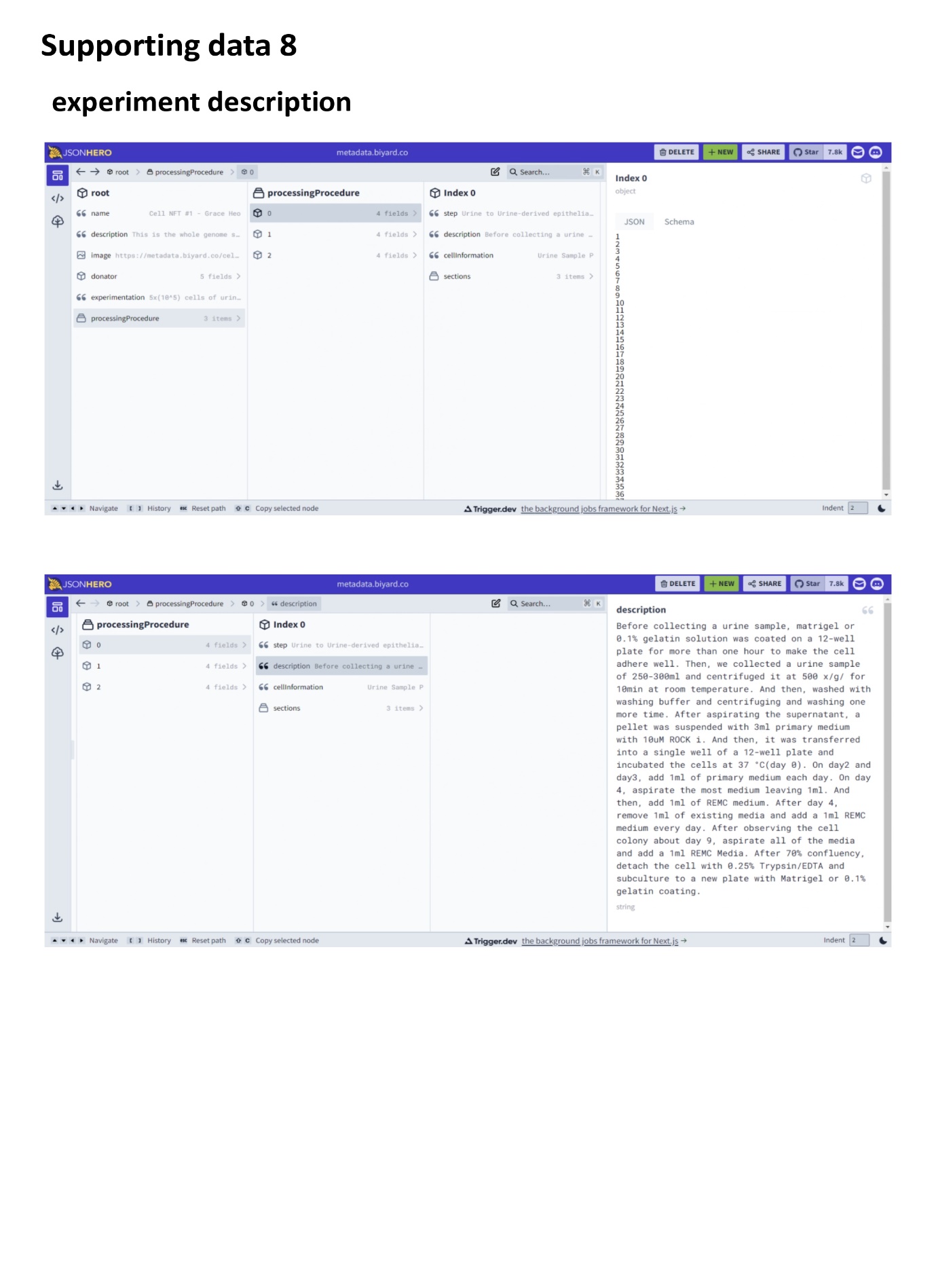


Supplementary Figure 8. The metadata of the actual issued Cell-NFT

**Supplementary Figure 9.** Cell-NFT Metadata example.

{
 "name": "Cell NFT #1 - Grace Heo",
 "description": "This is the whole genome sequencing of human. Whole genome sequencing(WGS) is a method of analyzing genetic information by reading the entire genome at once. Each person has a unique WGS. The variant information obtained through WGS is being used for disease-related gene discovery and personalized medicine research.",
 "image": "https://metadata.biyard.co/cell-nft/c1/wgs.png",
 "donator": {
 "name": "Grace Heo",
 "country": "Republic of Korea",
 "sec": "female",
 "birthday": "2002/08/24",
 "collectedAt": "2023/02/13"
 },
 "experimentation": "5x(10^5) cells of urine-derived cell was submitted to the Macrogen for sequencing. The samples were prepared according to the Illumina TruSeq Nano DNA library preparation guide or TruSeq DNA PCR-free library preparation guide. The libraries were sequenced using Illumina platform. Each sequenced sample is prepared according to the Illumina TruSeq DNA sample preparation guide to obtain a final library of 300-400 bp average insert size. For cluster generation, the library is loaded into a flow cell where fragments are captured on a lawn of surface-bound oligos complementary to the library adapters. Each fragment is then amplified into distinct, clonal clusters through bridge amplification. When cluster generation is complete, the templates are ready for sequencing. Illumina SBS technology utilizes a proprietary reversible terminator-based method that detects single bases as they are incorporated into DNA template strands. As all 4 reversible, terminator-bound dNTPs are present during each sequencing cycle, natural competition minimizes incorporation bias and greatly reduces raw error rates compared to other technologies. The result is highly accurate base-by-base sequencing that virtually eliminates sequence-context-specific errors, even within repetitive sequence regions and homopolymers. Each sequenced sample is prepared according to the Illumina TruSeq DNA sample preparation guide to obtain a final library of 300-400 bp average insert size.",
 "processingProcedure": [
 {
 "step": "Urine to Urine-derived epithelial cells(UDC)",
 "description": "Before collecting a urine sample, matrigel or 0.1% gelatin solution was coated on a 12-well plate for more than one hour to make the cell adhere well. Then, we collected a urine sample of 250-300ml and centrifuged it at 500 x/g/ for 10min at room temperature. And then, washed with washing buffer and centrifuging and washing one more time. After aspirating the supernatant, a pellet was suspended with 3ml primary medium with 10uM ROCK i. And then, it was transferred into a single well of a 12-well plate and incubated the cells at 37 °C(day 0). On day2 and day3, add 1ml of primary medium each day. On day 4, aspirate the most medium leaving 1ml. And then, add 1ml of REMC medium. After day 4, remove 1ml of existing media and add a 1ml REMC medium every day. After observing the cell colony about day 9, aspirate all of the media and add a 1ml REMC Media. After 70% confluency, detach the cell with 0.25% Trypsin/EDTA and subculture to a new plate with Matrigel or 0.1% gelatin coating. ",
 "cellInformation": "Urine Sample P",
 "sections": [
 {
 "name": "Passage 0",
 "culturing": {
 "location": "240A, College of Engineering, Yonsei university",
 "plate": "12well plate",
 "media": "REMC media",
 "date": {
 "start": "2023/02/13",
 "end": "2023/03/02"
 }
 },
 "images": [
 {
 "day": 10,
 "imageUrl": "https://metadata.biyard.co/cell-nft/c1/udc-p0-d10.png"
 },
 {
 "day": 12,
 "imageUrl": "https://metadata.biyard.co/cell-nft/c1/udc-p0-d12.png"
 },
 {
 "day": 15,
 "imageUrl": "https://metadata.biyard.co/cell-nft/c1/udc-p0-d15.png"
 }
 ]
 },
 {
 "name": "Passage 1~",
 "culturing": {
 "location": "240A, College of Engineering, Yonsei university",
 "plate": "60pi dish",
 "media": "REMC media",
 "date": {
 "start": "2023/03/02",
 "end": "2023/03/05"
 }
 },
 "images": []
 },
 {
 "name": "Passage 2",
 "culturing": {
 "location": "240A, College of Engineering, Yonsei university",
 "plate": "60pi dish",
 "media": "REMC media",
 "date": {
 "start": "2023/03/05",
 "end": "2023/03/07"
 }
 },
 "images": [
 {
 "day": 1,
 "imageUrl": "https://metadata.biyard.co/cell-nft/c1/udc-p2-d1.png"
 },
 {
 "day": 3,
 "imageUrl": "https://metadata.biyard.co/cell-nft/c1/udc-p2-d3.png"
 }
 ]
 }
 ]
 },
 {
 "step": "Urine-derived epithelial cells(UDC) to iPSC(induced Pluripotency Stem Cells)",
 "description": " Before reprogramming, matrigel or 0.1% gelatin solution was coated on a 35pi dish for more than one hour. Also, Yamanaka's factors hOCT4, hSK, and hUL were prepared with 1 μg/μL concentration. Prepared UDCs were detached with 0.25% Trypsin/EDTA and centrifuged at 300 x/g/ for 3min. After aspirating the supernatant, we counted cells for electroporation. 6*10^5 cells are required per electroporation. (based on a 100 μL tip). Collected cells were centrifuged at 300 x/g/ for 3min and the pellet was suspended with 100 μL R buffer per electroporation. After that, reprogramming plasmid hOCT4, hSK, and hUL was injected into cells with electroporation. The conditions for electroporation were set to 1650 V, 10 ms, and 3 pulses. After injecting the reprogramming plasmid, it was transferred into a 35pi dish with EM(day 0). REMC and iPSC culture medium E8 are used to culture, and the medium is changed every 24 hours. After 14 days pass, cell morphology changes from long morphology to round morphology. And then, by mechanical passaging, separate the part which has changed to round morphology which stands for iPSC. This mechanical passaging step needed ROCK inhibitor in E8 Medium for stable passaging. Stable iPSCs can be obtained after multiple mechanical passaging.",
 "cellInformation": "UDC Sample P_Passage2",
 "sections": [
 {
 "name": "Passage 0",
 "culturing": {
 "location": "240A, College of Engineering, Yonsei university",
 "plate": "6well plate",
 "media": "REMC media",
 "date": {
 "start": "2023/04/29",
 "end": "2023/05/21"
 }
 },
 "parameters": {
 "reprogrammingFactors": [
 "hOCT4",
 "hSK",
 "hUL"
 ],
 "delivery": {
 "method": "electroporation",
 "conditions": [
 {
 "name": "voltage",
 "unit": "V",
 "value": 1650
 },
 {
 "name": "duration",
 "unit": "ms",
 "value": 10
 },
 {
 "name": "pulse",
 "unit": "pulses",
 "value": 3
 }
 ]
 }
 },
 "images": [
 {
 "day": 7,
 "imageUrl": "https://metadata.biyard.co/cell-nft/c1/udcr-d7.png"
 },
 {
 "day": 14,
 "imageUrl": "https://metadata.biyard.co/cell-nft/c1/udcr-d14.png"
 },
 {
 "day": 16,
 "imageUrl": "https://metadata.biyard.co/cell-nft/c1/udcr-d16.png"
 }
 ]
 },
 {
 "name": "Passage 1",
 "culturing": {
 "location": "240A, College of Engineering, Yonsei university",
 "plate": "6well plate",
 "media": "E8 media",
 "date": {
 "start": "2023/05/17",
 "end": "2023/05/23"
 }
 },
 "parameters": {
 "reprogrammingFactors": [
 "hOCT4",
 "hSK",
 "hUL"
 ],
 "delivery": {
 "method": "electroporation",
 "conditions": [
 {
 "name": "voltage",
 "unit": "V",
 "value": 1650
 },
 {
 "name": "duration",
 "unit": "ms",
 "value": 10
 },
 {
 "name": "pulse",
 "unit": "pulses",
 "value": 3
 }
 ]
 }
 },
 "images": [
 {
 "day": 1,
 "imageUrl": "https://metadata.biyard.co/cell-nft/c1/ipsc-p1-d1.png"
 }
 ]
 }
 ]
 },
 {
 "step": "iPSC(induced Pluripotency Stem Cells) to Cardiomyocyte",
 "description": " Before seeding iPSCs, Matrigel or 0.1% gelatin solution was coated on a 12-well plate for more than one hour to make the cell adhere well. Then, prepared iPSCs were dissociated into a single cell with accutase for 8 minutes in an incubator. And then, collecting the detached cell and centrifuging at 200xg for 3min. And then, 0.5~1.5x10^6 cells per well were seeded in the 12well plate with E8 media which contains ROCK I.(Day -4) Until day 0, we changed the media and can obtain 85~90% confluency. On day 0, media was aspirated and RPMI with a B-27 supplement lacking insulin which contained 12μM CHIR99021 was added. The next day(day 1), the media was changed to RPMI with B-27 supplement lacking insulin. And day 3, 1ml RPMI with B-27 supplement lacking insulin medium which contained 5μm IWP2 was added to the conditioned medium. On day5, the media was changed to RPMI with a B-27 supplement lacking insulin. And then, on day7, the media was changed to RPMI with B-27 supplement with insulin. After that, media was changed per 3 days with RPMI with B-27 supplement with insulin.\n\nOnce the cardiomyocyte is produced, we can confirm that it beats like a heartbeat.",
 "cellInformation": "IPSC P147_PJH",
 "sections": [
 {
 "name": "Day -4 to Day 0",
 "culturing": {
 "location": "240A, College of Engineering, Yonsei university",
 "plate": "6well plate",
 "media": [
 "E8 with ROCKi",
 "E8 media"
 ],
 "date": {
 "start": "2023/03/21",
 "end": "2023/03/25"
 }
 },
 "images": [
 {
 "day": -3,
 "imageUrl": "https://metadata.biyard.co/cell-nft/c1/ipsc-d-3.png"
 },
 {
 "day": -2,
 "imageUrl": "https://metadata.biyard.co/cell-nft/c1/ipsc-d-2.png"
 }
 ]
 },
 {
 "name": "Day 0 to Day 19",
 "culturing": {
 "location": "240A, College of Engineering, Yonsei university",
 "plate": "6well plate",
 "media": [
 "RPMI w/B-27 w/o insulin",
 "RPMI w/B-27 w/ insulin"
 ],
 "date": {
 "start": "2023/03/25",
 "end": "2023/04/13"
 }
 },
 "parameters": {
 "CardiomyocyteInducingFactor": [
 "CHIR 99021",
 "IWP2"
 ]
 },
 "images": [
 {
 "day": 2,
 "imageUrl": "https://metadata.biyard.co/cell-nft/c1/ipsc-d2.png"
 },
 {
 "day": 12,
 "imageUrl": "https://metadata.biyard.co/cell-nft/c1/ipsc-d12.png"
 },
 {
 "day": 19,
 "imageUrl": "https://metadata.biyard.co/cell-nft/c1/ipsc-d19.png"
 }
 ]
 }
 ]
 }
 ]
}
